# High-Sensitivity PD-L1 Staining Using Clone 73−10 Antibody and Spatial Transcriptomics for Precise Expression Analysis in Non-Tumorous, Intraepithelial Neoplasia, and Squamous Cell Carcinoma of Head and Neck

**DOI:** 10.1007/s12105-025-01798-8

**Published:** 2025-05-20

**Authors:** Yuri Noda, Naho Atsumi, Takeo Nakaya, Hiroshi Iwai, Koji Tsuta

**Affiliations:** 1 Department of Pathology and Laboratory Medicine, Kansai Medical University Hospital, 2-3-1 Shin-machi, Hirakata, Osaka, 573-1191 Japan; 2 Department of Pathology, Kansai Medical University, 2-5-1 Shin-machi, Hirakata, Osaka, 573-1010 Japan; 3 Department of Otolaryngology, Head and Neck Surgery, Kansai Medical University Hospital, 2-3-1 Shinmachi, Hirakata, Osaka, 5731191 Japan

**Keywords:** Oral, Head and Neck squamous cell carcinoma, PD-L1, 73 − 10, Immune checkpoint inhibitor

## Abstract

**Purpose:**

While immune checkpoint inhibitors (ICIs) targeting the PD-1/PD-L1 axis have improved outcomes in head and neck squamous cell carcinoma (HNSCC), eligibility criteria based on immunohistochemistry (IHC) target PD-1 solely. We aimed to evaluate the PD-L1 (*CD274*) expression using highly sensitive clone 73 − 10 and spatial transcriptomics (ST) analysis to elucidate the role of PD-L1 in HNSCC and thus potentially expand the pool of eligible patients.

**Methods:**

Immunohistochemical staining of 73 − 10, CD3, CD4, and CD8 were performed in 94 HNSCC clinical samples along with paired adjacent squamous intraepithelial neoplasm (SIN) and normal oral mucosa (NOM) samples. The 73 − 10 positivity was evaluated using a tumor cell score ≥ 1%, and the results were analyzed against clinicopathological features including CD4^+^ and CD8^+^ tumor-infiltrating lymphocytes (TILs), and clinical outcomes. Furthermore, ST and PD-L1 related pathway analysis was performed in 6 paired HNSCC, SIN and NOM samples.

**Results:**

The 73 − 10 detected-PD-L1 positivity was high in HNSCC (79%) compared to SIN (10%) and NOM (3%). 73 − 10^+^ correlated with high CD4^+^ TILs, as well as the independent prognostic factor of OS, DSS, and PFS of HNSCC (all *p* < 0.05). ST analysis revealed that the upregulated distribution of *CD274* correlated with 73 − 10 positivity. Pathway analysis revealed a significant upregulation of *CD274* and C*D4* in HNSCC compared to SIN and NOM, and HIF-1α and IFN-γ as key regulators of PD-L1 expression in HNSCC.

**Conclusion:**

Clone 73 − 10 is a relatively suitable candidate for identifying patients with PD-L1 expression eligible for ICI therapy. It demonstrates high sensitivity in detecting PD-L1 (*CD274*) in HNSCC, offering immunological and prognostic insights.

**Supplementary Information:**

The online version contains supplementary material available at 10.1007/s12105-025-01798-8.

## Introduction

Head and neck squamous cell carcinoma (HNSCC) is the seventh most common cancer worldwide [1]. Its 5-year survival rate is 60%; however, late-stage cases do tend to have a worse prognosis [1]. Currently, immune checkpoint inhibitors (ICIs), such as pembrolizumab and nivolumab, blocking the PD-1/PD-L1 axis, have been approved for the treatment of patients with HNSCC who have disease progression or failed platinum-based chemotherapy [2–5]. Although ICIs have improved outcomes in some patients, approximately 60% remain ineligible for treatment [5–8], and only 15% of those who respond to ICI treatment experience further potential for improvement in patients pre-selected based on PD-L1 expression [9]. To address these challenges and broaden the pool of eligible patients, innovative evaluation methods using relatively sensitive antibody clones closely aligned with the mRNA expression profiles of ICI target antigens are urgently needed [5].

Immunohistochemistry (IHC) is a commonly employed companion diagnostic tool for assessing patient eligibility for ICI therapies. Recent studies have demonstrated that the 73 − 10 clone exhibits superior sensitivity in detecting PD-L1 expression than the five FDA-approved IHC clones [10, 11]. ICIs, such as avelumab, when used to select patients based on 73 − 10 IHC, have shown promise in prolonging survival in patients with non-small cell lung carcinoma [12], urothelial carcinoma [13], and metastatic breast cancer [14]. In HNSCC, a phase I trial (NCT02517398) of bintrafusp alfa, a bifunctional fusion protein targeting TGF-β and PD-L1, revealed outcomes equivalent to those of pembrolizumab and nivolumab treatments. In this study, patients were categorized based on 73 − 10 IHC, using a tumor cell (TC) score threshold of 1% (TC-positive ≥ 1%, TC-negative < 1%) [15]. However, no clinicopathological studies have yet examined in detail the role of PD-L1 (*CD274*) and 73 − 10 IHC expression in patients with HNSCC.

Furthermore, the current companion diagnostics of HNSCC target the PD-1 antigen only (clones 22 C-3 and 28 − 8) [16–19]. Therefore, evaluating the PD-1/PD-L1 axis from a novel perspective—specifically by assessing PD-L1 expression—may expand the pool of potentially eligible patients. Furthermore, to explore the clinical applicability of this approach, IHC-detected PD-L1 protein expression must be validated by correlating it with corresponding mRNA expression (*CD274*) and assessing relevant signaling pathway activity.

In this study, we aimed to evaluate PD-L1 and its mRNA (*CD274*) expression using 73 − 10 IHC and Visium Spatial Transcriptomics (ST) assay—a technique that preserves tissue architecture and links mRNA expression to its original location [20]—to investigate its potential to expand the pool of eligible patients for ICI treatment. In total, 100 patients with progressive HNSCC were included, and the correlation between 73 − 10 expression, mRNA (*CD274*) expression, and their distribution was analyzed. Furthermore, we assessed the clinicopathological features and outcomes of these patients; in addition, we performed pathway analysis to further elucidate the prognostic value of 73 − 10 expression in HNSCC. This study offers a more accurate and reliable method for selecting patients with HNSCC for ICI treatment.

## Materials and Methods

### Patients for Clinicopathological Analysis

This retrospective study included 94 patients with progressive HNSCC for clinicopathological IHC analysis who underwent surgical resection at the Department of Otorhinolaryngology, Head and Neck Surgery, Kansai Medical University Hospital, between January 2009 and December 2024. Patients diagnosed with progressive HNSCC at pathological stages pT3 or pT4 and those with non-decalcified formalin-fixed paraffin-embedded (FFPE) blocks were included in this study. This study was approved by the Institutional Review Board (approval number: 2023024). The detailed demographic and clinicopathological characteristics of the patients are provided in Online Resources 1 and 2.

### Construction of Tissue Microarrays

For clinicopathological analysis, Tissue Microarrays (TMAs) were constructed from 1040 cores, with four cores each from 62 normal oral mucosa (NOM) samples, 94 squamous intraepithelial neoplasm (SIN) samples including mild to severe dysplasia, and 94 HNSCC samples obtained from surgically resected FFPE tissue samples of 94 patients with HNSCC (tissue specimens for TMA cores of HNSCC and SIN were obtained from 94 patients with HNSCC; however, NOM specimens were obtained from 62 of these 94 HNSCC patients). In the present study, we excluded the carcinoma in situ (CIS) from analysis to avoid confusion between CIS and SCC, and considered the genetic differences between dysplasia and CIS. Four HNSCC cores were collected from different invasive areas: two from superficial invasive areas (depth of invasion ≤ 5 mm) and two from deeper invasive fronts (depth of invasion > 6 mm). Each FFPE tissue block was sampled with 2.0-mm cores using a tissue-arraying instrument (Azumaya Corporation, Tokyo, Japan). All selected cores contained at least 100 epithelial cells.

### Histopathological and Clinicopathological IHC Analyses Using Tissue Microarray

Histopathological analysis was performed on all tissue cores using hematoxylin-eosin (HE)-stained slides made from TMA to evaluate pathological factors within the tumor microenvironment, including desmoplastic reaction (DR), tumor budding (BUD), and tumor-infiltrating lymphocytes (TILs), as described previously [21–23]. For each case, the highest values for each parameter were recorded from each group including NOM, SIN and HNSCC, as they were the most representative of the immune components when present. Additional clinicopathological data were collected from the hospital medical records.

For IHC analysis, tissue sections were incubated with antibodies against 73 − 10 (pre-diluted; Leica Biosystems, Newcastle Upon Tyne, UK), CD3 (PS1, pre-diluted; Nichirei Bioscience, Inc., Tokyo, Japan), CD4 (1F6, pre-diluted; Nichirei Bioscience, Inc.), and CD8 (G2B10, 1:20000; Proteintech Group, Inc. IL, USA). The 73 − 10 antibody was visualized using Leica Bond-III (Leica Biosystems, Melbourne, Australia) and Bond Polymer Refine Detection systems (Leica Biosystems) according to the manufacturer’s instructions. For CD3, CD4, and CD8, antigen retrieval was performed using ethylenediaminetetraacetic acid or citrate buffer at 95 °C for 1 h, followed by detection using a Histofine Simple Stain MAX-PO^®^ polymer detection system (#NIC-414131 F; Nichirei Bioscience Inc.), and visualization using diaminobenzidine.

The expression of 73 − 10 was evaluated based on the TC score, traditionally defined using the invasive carcinoma components for both the numerator and denominator. However, in the present study, the non-neoplastic epithelium for NOM and the dysplastic components for SIN were used while maintaining the original method for invasive carcinoma components in HNSCC. The TC score was calculated as the percentage of viable epithelial cells exhibiting membrane staining, with TC ≥ 1% considered positive and TC = 0 considered negative [15–18]. The percentage of CD4^+^ and CD8^+^ TILs was measured and classified as low (< 20%) or high (≥ 20%) based on CD3^+^ areas. Core with low CD4 and CD8 expression were classified as low immune-active, whereas all others were classified as highly immune-active. TC and TILs were assessed in all cores, with the highest values among the four cores each NOM, SIN, and HNSCC were recorded. For assessing the utility of TC in biopsies, the TC of HNSCC was recorded from both the superficial core and the invasive front core.

### Visium Spatial Transcriptomic Analysis

Visium ST analysis (103 Genomics, Pleasanton, CA, USA) was performed on 18 TMA cores from an additional six patients with progressive HNSCC [sample 1 (S1) to sample 6 (S6)] to investigate the spatial landscape of gene expression. These patients were selected based on previously outlined criteria, with the additional condition that their FFPE blocks had been prepared within the previous month to minimize mRNA degradation. Three 2 × 2 mm cores were collected from each patient: one from HNSCC, one from SIN, and one from NOM.

Raw read count matrices were normalized to transcripts per kilobase of exome per million mapped reads. Differential gene expression analysis was performed using single-cell RNA sequencing (scRNA-seq) datasets to compare expression patterns across samples from six patients (S1–S6) and three clusters. The epithelium was categorized into four clusters: NOM, SIN, HNSCC, and non-evaluable epithelium (excluded epithelium from ICI IHC assessment where apoptosis or necrosis marker positive area, or without nuclei or with necrosis and hemorrhagic area) (categorization is summarized in Online Resource 3). The analysis was performed using the Seurat package (v4.1.1) following the steps outlined in Online Resource 3. After processing the transcriptomic data, differentially expressed genes (DEGs) from 18 samples (six NOM, six SIN, and six HNSCC) were normalized, and differential gene expression analysis was conducted using the FindMarkers function. A log-fold change threshold was set to 0.25, and *p*-values < 0.05, adjusted using Bonferroni correction, were applied, with a minimum expression percentage (min.pct) of 0.1, to identify the significant DEGs in each cluster. The correlation between mRNA expression and 73 − 10 IHC positivity (TC ≥ 1%) was identified at log_2_ fold change ≥ 0.25 and p-value < 0.05, compared to other clusters in the same cases. Subsequently, KEGG pathway analyses of DEGs between NOM vs. SIN and HNSCC, SIN vs. NOM and HNSCC, and HNSCC vs. NOM and SIN were performed specifically for PD-L1 expression and PD-1 checkpoint pathway in cancer - homo sapiens (human) (hsa05235) and T-cell receptor signaling pathways (hsa04660). Furthermore, hsa04660 of HNSCC was compared with that of NOM (https://www.genome.jp/kegg/; accessed 2024/10/19).

### Statistical Analyses

Correlations between clinicopathological features and 73 − 10 TC expression levels were determined using Fisher’s exact test. The cutoff value for tumor immune activity was calculated as the area under the curve against overall survival (AUC) against OS. A multivariate logistic regression Cox hazard model was constructed to assess the relationship between the predictor variables. If only two factors remained statistically significant, a bivariate analysis was performed. Log-rank tests were used to evaluate OS, disease-specific survival (DSS), and recurrence-free survival (RFS). The association between 73 − 10 TC positive and *CD274* mRNA upregulation was examined using the Pearson correlation coefficient. All statistical analyses were performed using the IBM SPSS Statistics software (v20.0; IBM Corp., Armonk, NY, USA). The significance of mRNA expression was set at log_2_ fold-change > 0.25 and *p* < 0.05, and the significance of other analyses was set at *p* < 0.05.

## Results

### Demographic and Clinicopathological Characteristics of Patients with 94 HNSCC

The 94 patients comprised 59 males and 35 females, aged between 30 and 87 years (median: 69 years; mean: 68.7 ± 10.9 years) located in the buccal mucosa (*n* = 6), gingiva (*n* = 26), floor of the mouth (*n* = 1), and tongue (*n* = 61). The survival time of the patients ranged from 1 to 180 months (median: 42 months; mean: 47.7 ± 39.9 months). During the follow-up period, 41 patients, 28 of whom succumbed to HNSCC, died.

A slight predominance of low TIL was revealed (55%, 52/94) compared to high TIL (45%, 42/94). Notably, the CD4^+^ helper T cell response was more prominent than the CD8^+^ cytotoxic T cell response [CD4^+^ TILs: low 55% (52/94), high 45% (42/94); CD8^+^ TILs: low 65% (61/94), high 35% (33/94)]. Overall, HNSCC samples exhibited a predominantly low immune-active status [low immune active: 79% (74/94) vs. high immune active: 21% (20/94)], suggesting T cell exhaustion, particularly of CD8^+^ T cells, and potential sensitivity to ICI therapy.

### Expression Levels of 73 − 10 in NOM, SIN, and HNSCC Cores

**Fig. 1:**
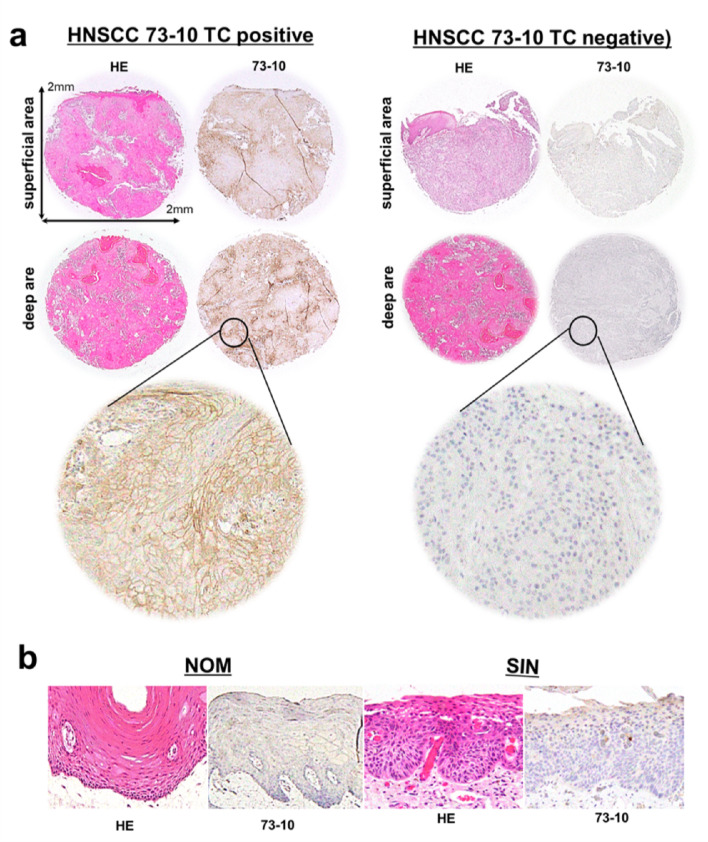
73 − 10 expression in NOM, SIN, and HNSCC. The 73 − 10 staining indicates the absence of NOM (**a**), is focally observed in SIN (**b**), and is positive (TC ≥ 1%) in both the superficial and deeper cores of HNSCC (b, left), and both negatives (TC < 1) in HNSCC cores (b, right). HE, hematoxylin-eosin staining; NOM, normal mucosa; SIN, squamous intraepithelial neoplasm; HNSCC, head and neck squamous cell carcinoma

Evaluation of TC in NOM, SIN, and HNSCC cores revealed that the number of positive cases of 73 − 10 TC in NOM, SIN, and HNSCC was 3% (2/62), 10% (9/94), and 79% (74/94), respectively (Fig. 1a, b; Table 1). To analyze heterogeneous PD-L1 expression in tumors, the expression distribution was examined in superficial and deep invasive front areas. Among the 74 PD-L1-positive HNSCC cases, the majority (88%, 65/74) showed positivity in both areas, while a smaller proportion were positive only in the superficial (7%, 5/74) or deep invasive front areas (5%, 4/74).

**Table 1:** Positive expression cases of 73 − 10 TC in the area of HNSCC cores

	Expression areas		positive (%)
NOM	-		**3% (2/62)**
SIN	-		**10% (9/94)**
HNSCC	superficial/deep core: (+/+)	88% (65/74)	**79% (74/94)**
	superficial/deep core: (+/-)	7% (5/74)	
	superficial/deep core: (-/+)	5% (4/74)	

NOM, normal oral mucosa; SIN, squamous intraepithelial neoplasm; HNSCC, head and neck squamous cell carcinoma

### Clinicopathological Analysis of 73 − 10 Expression

The clinicopathological analysis in 94 patients with HNSCC revealed significant associations of 73 − 10 positivity with high CD4^+^ TILs expression, pDOI ≥ 10 mm, and the presence of lymphovascular invasion (all, *p* < 0.05), but no significant associations with the expression of TILs, CD8^+^ TILs, immune-active status, and other clinicopathological features (*p* > 0.05, Table 2).

**Table 2:** Association of TC for 73 − 10 IHC clone and clinicopathological features of 94 patients with HNSCC

		TC for 73 − 10 (HNSCC, *n* = 94)		
Clinicopathological features		Negative	Positive	*p*-value
Differentiation	well/moderately	12 (22%)	42 (78%)	1
	poorly	8 (20%)	32 80%)	
**Ly**	Negative	8 (42%)	11 (58%)	***0.02***
	Positive	12 (16%)	63 (84%)	
V	Negative	5 (33%)	10 (67%)	0.3
	Positive	15 (19%)	64 (81%)	
Pn	Negative	5 (25%)	15 (75%)	0.76
	Positive	15 (20%)	59 (80%)	
Invasion pattern	YK-1,2	3 (33%)	6 (67%)	0.4
	YK-3,4	17 (20%)	68 (80%)	
**pDOI**	< 10 mm	5 (50%)	5 (50%)	***0.03***
	≥ 10 mm	15 (18%)	69 (82%)	
pN	pN0,1	12 (27%)	33 (73%)	0.31
	pN2,3	8 (16%)	41 (84%)	
pENE	absence)	17 (25%)	52 (75%)	0.26
	presence	3 (12%)	22 (88%)	
BUD	low	10 (24%)	31 (76%)	0.61
	high	10 (19%)	43 (81%)	
DR	mature	4 (17%)	20 (83%)	0.77
	immature	16 (23%)	54 (77%)	
TILs	low	13 (21%)	49 (79%)	0.23
	high	7 (22%)	25 (78%)	
**CD4**^**+**^TILs	low	17 (33%)	35 (67%)	***< 0.001***
	High	3 (7%)	39 (93%)	
CD8^**+**^TILs	low	14 (22%)	47 (78%)	0.79
	High	6 (18%)	27 (82%)	
Immune active	low	18 (24%)	56 (76%)	0.23
	High	2 (10%)	18 (90%)	

BUD, tumor budding; DR, desmoplastic reaction; HNSCC, head and neck squamous cell carcinoma; Ly, lymphovascular invasion; V, vascular invasion; Pn, perineural invasion; pDOI, pathological depth of invasion; pN, pathological lymph node metastasis; pENE, pathological extranodal extension; BUD, budding; DR, desmoplastic reaction; TILs, tumor-infiltrating lymphocytes. **Bold**: *p* value < 0.05

### Prognostic Values of TC for 73 − 10 in Patients with HNSCC

**Fig. 2:**
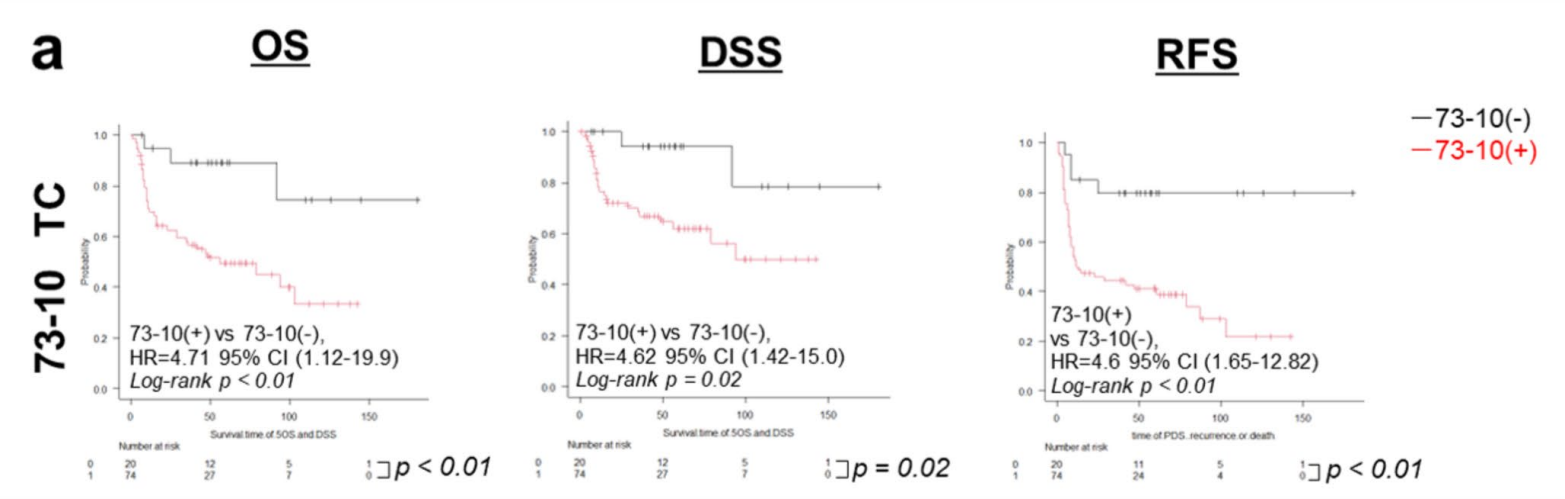
OS, DSS, and RFS of HNSCC evaluated using 73 − 10 detected PD-L1. OS, overall survival; DSS, disease-specific survival; RFS, recurrence-free survival; HNSCC, head and neck squamous cell carcinoma; TC, tumor cell score; CI, confidence interval

The cox hazard test showed that 73 − 10 positive is the most powerful prognostic indicator of OS (hazard ratio [HR] 5.13, 95% confidence interval [CI] 1.56–17.04, *p* = 0.007), DSS (5.46; 1.26–23.61, 0.023), and RSS (4.49, 1. 60–12.57, 0.04) in patients with progressive HNSCCs compared to age, BUD, CD4^+^ TILs, CD8^+^ TILs, DR, immune activity, pDOI > 10 mm, pENE, and pN (Table 3). The log-rank test revealed that patients with progressive HNSCC showing 73 − 10 positive had significantly worse OS, DSS, and RFS than those with 73 − 10 negative tumors (all *p* < 0.05, Fig. 2). Moreover, low CD4^+^ T-cell infiltration, low CD8^+^ T-cell infiltration, low numbers of TILs, and immune-desert status were associated with poor prognosis in terms of OS, DSS, and RFS (log-rank test, all *p* > 0.05; Online Resource 4).

**Table 3:** Cox hazard ratio of OS, DSS, and RFS in HNSCC

	OS			DSS			RFS		
Multivariate	HR	95%CI	*p*-value	HR	95%CI	*p*-value	HR	95%CI	*p*-value
Age > 64	1.34	0.65–2.74	0.43	1.60	0.62–4.10	0.33	1.47	0.76–2.82	0.25
BUD high	1.55	0.62–3.87	0.34	1.18	0.40–3.52	0.76	1.34	0.64–2.77	0.44
CD4 + TILs low	1.61	0.46–5.63	0.46	2.17	0.43–11.04	0.35	0.86	0.28–2.65	0.79
CD8 + TILs low	1.25	0.44–3.56	0.68	0.85	0.18-4.00	0.84	0.91	0.39–2.12	0.82
DR immature	1.17	0.43–3.16	0.76	1.74	0.47–6.46	0.41	1.32	0.58-3.00	0.51
Immune active low	1.45	0.32–6.43	0.63	2.62	0.33–20.51	0.36	2.21	0.59–8.32	0.24
pDOI > 10 mm	0.61	0.18–2.04	0.42	0.38	0.08–1.71	0.20	0.67	0.23–1.97	0.47
**pENE presence**	**3.17**	**1.30–7.73**	**0.01**	2.82	0.91–8.70	0.07	**2.21**	**1.01–4.84**	**0.05**
pN presence	1.50	0.59–3.81	0.40	2.36	0.70–7.94	0.17	1.14	0.52–2.48	0.74
**TC for 73 − 10 ≥ 1**	**8.66**	**2.33–32.27**	**0.001**	**14.48**	**2.58–81.27**	< 0.001	**5.71**	**1.95–16.70**	**0.001**
**Bivariate**	HR	95%CI	*p*-value	HR	95%CI	*p*-value	HR	95%CI	*p*-value
pENE presence	4.23	2.22–8.07	< 0.001	4.78	2.19–10.43	< 0.001	2.39	1.32–4.31	0.004
**73 − 10 positive (TC ≥ 1%)**	**5.16**	**1.56–17.04**	**0.007**	**5.46**	**1.26–23.61**	**0.023**	**4.49**	**1.60-12.57**	**0.004**

OS: overall survival, DSS: disease-specific survival, RFS: recurrence-free survival, HR, Hazard ratio, CI: confidence interval, BUD: budding, TILs: tumor-infiltrating lymphocytes; DR, desmoplastic reaction; pDOI: pathological depth of invasion, pENE: pathological extranodal extension, pN: pathological lymph node metastasis; TC, tumor cell score. **Bold**: *p* value < 0.05

### Associations of 73 − 10 Expression and *CD274* mRNA and Protein Distributions in HNSCC Tissue

*CD274* (PD-L1) mRNA expression was further examined on Visium ST spots from NOM, SIN, and HNSCC samples to determine whether 73 − 10 IHC expression reflected underlying molecular features.

**Fig. 3:**
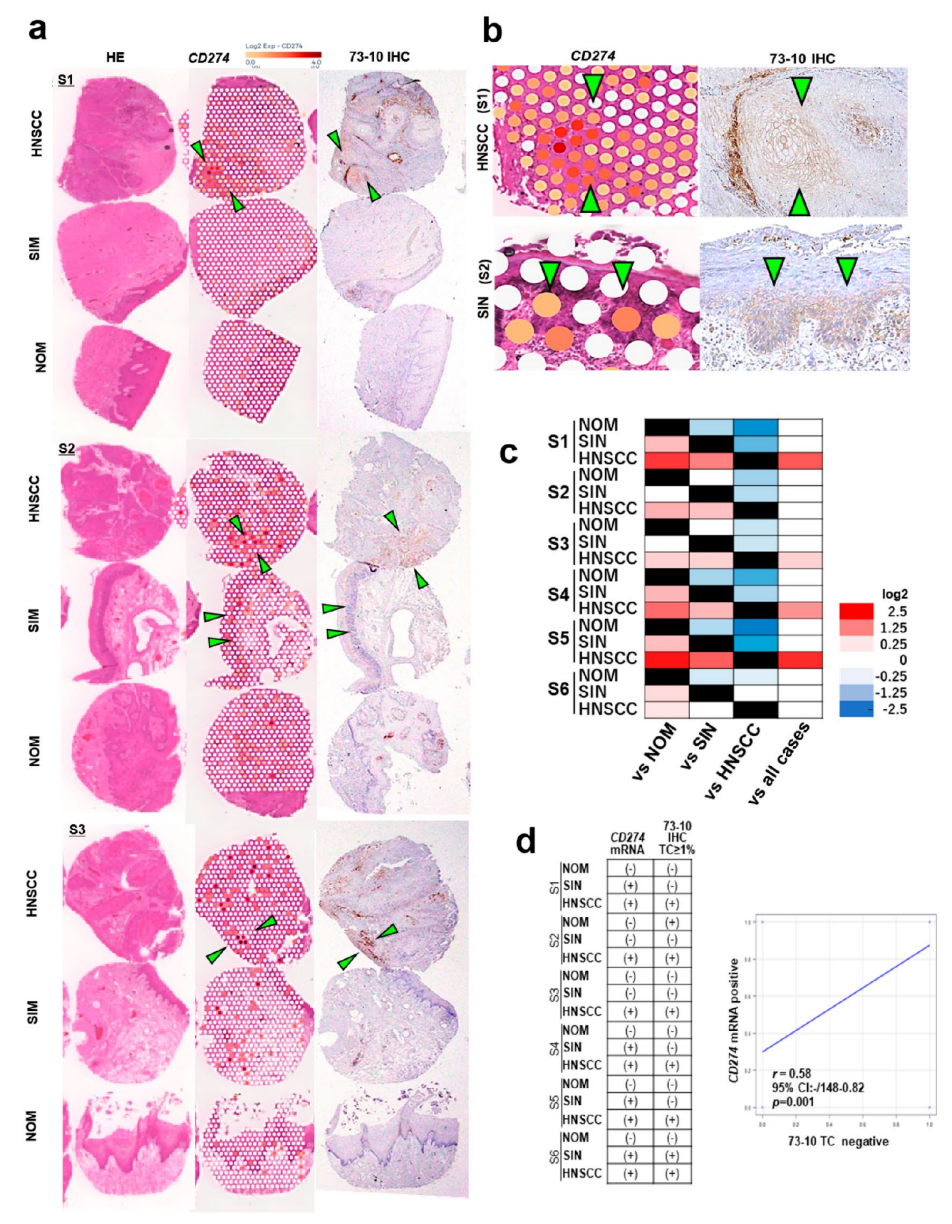
73 − 10 detected PD-L1 and *CD274* mRNA expression. Verification of PD-L1 expression at mRNA and protein levels in NOM, SIN, and HNSCC (**a**, **b**); green arrows indicate PD-L1 and *CD274* mRNA overexpression area. PD-L1 and *CD274* mRNA expression in all six cases (**c**). Correlation coefficient between 73 − 10 detected PD-L1 and *CD274* mRNA expression (**d**). PD-L1, Programmed death-ligand 1; HE, hematoxylin and eosin; IHC, immunohistochemistry; HNSCC, head and neck squamous cell carcinoma; SIN, squamous intraepithelial neoplasia; NOM, normal oral mucosa; S, sample; CI, confidence interval

The expression of *CD274* mRNA and the 73 − 10 IHC staining are shown in Fig. 3a, b, and their overexpression distributions matched. *CD274* was significantly upregulated in neoplasms compared to NOM groups, especially in HNSCC groups (log_2_ fold > 0.25 and *p* < 0.05, Fig. 3a, b). Furthermore, it was significantly upregulated in core of HNSCC (100%) and SIN (66%) compared to that in the paired NOM (Fig. 3c). Five samples (88%) exhibited the highest *CD274* expression in HNSCC cores among the three groups (Fig. 3c and Online Resource 5). Immunohistochemically, a higher 73-10-positive expression frequency was observed in HNSCC (100%) than in SIN and NOM (each 16%), which was consistent with the mRNA expression results. The coefficients of correlation between the negative or positive status for *CD274* and 73 − 10 were 83% for NOM, 50% for SIN, and 100% for HNSCC, with a significant correlation (*r* = 0.58, 95% CI: 0.148–0.82, *p* = 0.01, Fig. 3d).

### Pathway Analysis of PD-L1 in HNSCC, SIN, and NOM

Based on the correlation between 73 − 10 IHC and *CD274* expression in HNSCC, SIN, and NOM, pathway analysis was performed using Visium-derived DEGs to elucidate the mechanisms driving poor prognosis in PD-L1-positive HNSCC cases.

**Fig. 4:**
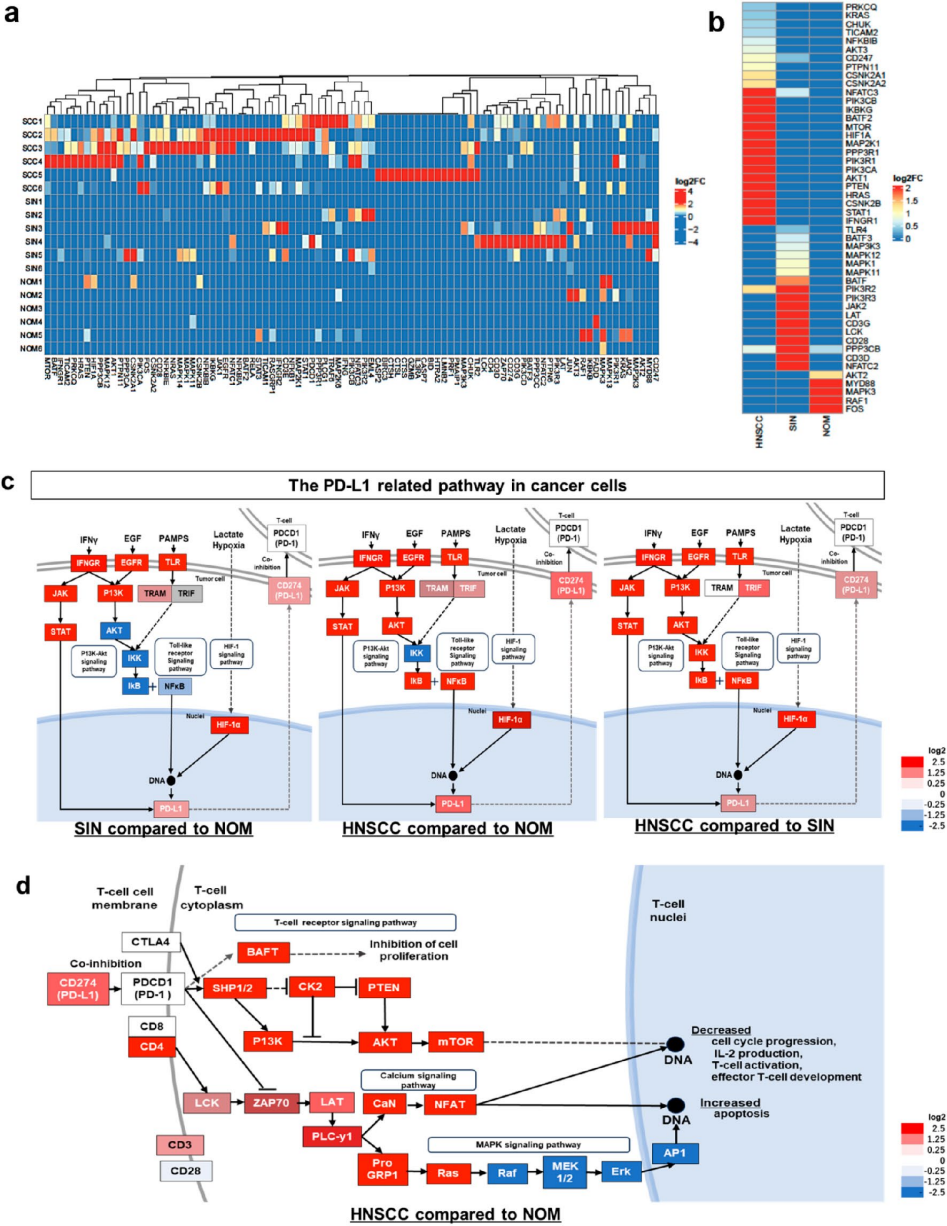
Gene expression in PD-L1-related and T-cell receptor pathways in cancer cells. Log_2_ enhanced heat maps of PD-L1 related genes in six HNSCC, six SIN, and six NOM samples (**a**). Comparison of the three groups (**b**). PD-L1-related pathways in cancer cells compared to NOM, SIN, and HNSCC (**c**). T-cell receptor signaling in HNSCC compared to NOM (**d**). PD-L1, Programmed death-ligand 1; HNSCC, head and neck squamous cell carcinoma; SIN, squamous intraepithelial neoplasia; NOM, normal oral mucosa; HIF-1α, hypoxia-inducible factor-1 alpha; IFN-γ, interferon-gamma

A total of 94 DEGs were detected in 18 cores (6 cores of NOM, 6 cores of SIN, and 6 cores of HNSCC) obtained from the PD-L1-related signaling pathway in cancer cells (hsa05235). Pathway analysis revealed that most genes included in has05235 were upregulated in HNSCC (Fig. 4a, Online Resource 6). Among the 49 significant DEGs in the NOM, SIN, and HNSCC groups (Fig. 4b, Online Resource 6), the hypoxia-inducible factor-1 alpha (HIF-1α) and interferon-gamma (IFN-γ) pathways were significantly upregulated in HNSCC compared to NOM and SIN, with further upregulation observed relative to SIN (Online Resource 7). Other signaling pathways, such as the toll-like receptor, PI3K-Akt, and MAPK pathways, were also activated in HNSCC and SIN; however, some downstream genes were downregulated compared with NOM.

### Pathway Analysis of PD-L1 Check Point Pathway in T-cells Compared To HNSCC and NOM

In the epithelium of HNSCC and SIN compared to NOM, Hsa05235 analysis showed upregulation of *CD274* (PD-L1) and its related pathways; however, no upregulation of *PDCD1* (PD-1) on T-cells was observed. Therefore, the T-cell receptor signaling pathway (hsa04660) was further analyzed, revealing that neither *PDCD1* (PD-1) nor *CTLA4*, the ICIs target ligands, were upregulated in HNSCC, as also confirmed by ST (Fig. 4d, Online Resource 7) and SIN (data not shown) compared with NOM. However, the downstream signaling of *PDCD1*, including genes such as *SHIP1/2*, *BATF*, and *PI3K*, was upregulated, suggesting that pathways beyond *PDCD1* may also contribute to T cell suppression.

Among the T-cell markers, only the expression of *CD4* was significantly upregulated in HNSCC compared to that in NOM, and its downstream pathway, the calcium signaling pathway, was also upregulated. However, no significant differences were observed in the expression levels of *CD3*, *CD8*, or *CD28*. These results suggest that PD-L1 may be a more effective ICIs target than PD-1 and CTLA4, with CD4^+^ T-cells playing a key role in the tumor immune response in progressive HNSCC.

## Discussion

In this study, PD-L1 expression in HNSCC was evaluated using the 73 − 10 IHC clone, revealing that 79% of cases were PD-L1 positive (TC ≥ 1%) and associated with poor prognosis owing to low CD4^+^ T-cell infiltration. This PD-L1 upregulation may have induced by activation of HIF-1α and IFNγ cascades during HNSCC carcinogenesis. Notably, significant 73-10-detected PD-L1 protein expression was observed in the tumor cells of invasive HNSCC compared to the epithelium of SIN and NOM, with relatively uniform expression throughout the tumor and high concordance with *CD274* mRNA expression in terms of both spatial distribution and expression levels. However, neither *PDCD1* (PD-1) nor *CTLA4* were upregulated. These novel aspects collectively support the clinical utility of evaluation using the 73 − 10 IHC-detected PD-L1 expression with CD4^+^ T-cells playing a key role in the tumor immune response in progressive HNSCC.

First, we evaluated the potential of 73 − 10 IHC to detect whether PD-L1 expression could be used as a target of ICIs and expand the pool of eligible patients with HNSCC for ICIs. The 73 − 10 TC ≥ 1% was observed in 79% of patients with HNSCC, which consistent with previous study, reporting 73 − 10 TC ≥ 1% in 78% of patients with progressive HNSCC [15]. These positive rate notably higher than the 40–60% observed in previous evaluations of HNSCC using other PD-L1/PD-1 clones [16–18]. On lung and bladder cancers assessed with 73 − 10 clone showed higher sensitivity than other clones, likely because of its ability to recognize the intracellular domain of PD-L1, whereas other clones target the extracellular domain [9]. Furthermore, a high concordance in PD-L1 expression between superficial and deep tumor cores was observed, in line with a previous report on the high concordance between biopsy and resection samples [19]. These findings suggest that the 73 − 10 TC ≥ 1% evaluation method may offer a highly sensitive and specific approach to identifying patients who could benefit from ICIs. In other words, with the potential to address concerns about tumor heterogeneity associated with sampling sites, this method could improve biopsy suitability and help expand the pool of patients with HNSCC eligible for immunotherapy.

Moreover, the 73 − 10 TC ≥ 1% evaluation method demonstrated strong prognostic value. The 73 − 10 TC ≥ 1% status was the most significant independent prognostic factor for OS, DSS, and PFS in patients with HNSCC, outperforming the other clinicopathological features. These findings are consistent with results from a Phase I clinical trial [15], which also used a 73 − 10 TC ≥ 1% cutoff to evaluate the outcomes. Furthermore, the association between 73 − 10 positivity and high CD4^+^ TILs in progressive HNSCC aligns with the mRNA expression data related to the PD-L1 signaling pathway. In some cancers including HNSCC, intratumoral CD4^+^ T cells exhibit cytotoxic phenotypes capable of directly killing cancer cells, similar to CD8^+^ T-cells, while performing helper functions [24]. However, the HNSCC cohort exhibited a predominantly low immune-active status in most samples (80%), and no correlation was found between CD8^+^ T cell infiltration and PD-L1 expression. Despite this overall low immune activity, this study, fortified by a large sample size comparable to or exceeding those of previous reports, revealed through signaling pathway analysis a strong association between CD4^+^ TILs and PD-L1 expression. Furthermore, the minimal influence of tumor heterogeneity on PD-L1 is reinforced. These results highlight the novel insight that CD4^+^ T cells may play a more critical immunomodulatory role in HNSCC.

The findings also revealed that 73 − 10 IHC expression and distribution corresponded to *CD274* (PD-L1) mRNA expression, showing upregulation in the epithelium of invasive HNSCC compared to NOM and SIN. This suggests their involvement in PD-L1 expression during carcinogenesis of progressive HNSCC. Previous studies examining the relationship between PD-L1 mRNA and protein expression in various cancer cell lines, including HNSCC cells, have shown similar results [25]. Furthermore, the present study provides novel evidence that 73 − 10 IHC detecting PD-L1 expression corresponds to that of mRNA at the spatial distribution level, with upregulation observed in the order of HNSCC, SIN, and NOM in all six cases. These findings reveal that 73-10-detected PD-L1 plays an important role in the tumor microenvironment during the malignant transformation process and that 73 − 10 IHC as a visually accessible method for assessing PD-L1-related pathway activation.

Pathway analysis revealed that the HIF-1α and IFNγ pathways are particularly critical for PD-L1 upregulation in HNSCCs. Furthermore, neither *PDCD1* (PD-1) nor *CTLA4*, the ICI target ligands, were upregulated. These results suggest that PD-L1 may be a more effective ICI target than PD-1 and CTLA4. In multiple cancers, including HNSCC, HIF-1α translocates to the nucleus to promote malignant cell survival [26, 27]. Additionally, T-cell products, including IFN-γ, activate the IFN-γ signaling pathway, which further upregulates PD-L1 expression in tumor cells [28]. The poor prognostic factors of HNSCC include severe hypoxia, elevated IFN-γ levels, and a suppressed immune response [28, 29]. The findings of the present study align with these observations, suggesting that HIF-1α and IFNγ pathways may contribute to the poor prognosis of 73 − 10 IHC-positive HNSCC. In addition, *PDCD1* (PD-1) and *CTLA4* were not significantly upregulated in HNSCC compared with NOM and SIN, despite the upregulation of *CD274* (PD-L1). Similar observations of PD-1 expression in SIN and HNSCC compared to NOM have also been reported [30]. In patients with HNSCC, IHC using 22 C-3 and 28 − 8 clone are approved as companion diagnostic tools for PD-1 expression. Conversely, anti-CTLA-4 antibodies, which target activated T cells, especially CD4 ^+^ regulatory T-cells, have not yet been approved for the treatment of HNSCC [31]. Considering that PD-L1 inhibition is an established approach in clinical settings, our findings suggested that PD-L1 targeting therapy or a combination therapy targeting HIF-1α and IFN-γ might represent a promising novel strategy for treating HNSCC.

This study has several limitations. First, the single-institution design and lack of post-treatment HNSCC samples limit the generalizability of our findings. Additionally, excluding HPV-associated HNSCCs may limit the applicability of our results. ICIs are more effective in HPV-associated HNSCC owing to higher PD-L1 expression from HPV oncoproteins and T-cell exhaustion [32–35], suggesting PD-L1 evaluation with the 73 − 10 clone is particularly useful for this subtype. Despite the multi-core TMA approach, 10% of HNSCC cases still exhibited heterogeneous 73 − 10 staining, highlighting the need to consider re-biopsy in cases with negative results. Furthermore, to enhance generalizability and clinical applicability, 73 − 10 expression should be evaluated using the Combined Positive Score, as this scoring method is standard for companion IHC diagnostics in HNSCC. We are preparing a separate manuscript that includes these results for publication in the near future. Future studies should include a larger cohort of patients, both with and without prior treatment, to compare treatment outcomes between the evaluation systems using 73 − 10 and other PD-L1 clones. Future studies are needed to clarify the potential confounding factor of patients with high PD-L1 expression receiving ICI therapy and experiencing improved outcomes between the evaluation systems including other PD-L1 clones and other methods (e.g., CPS, cut off: 0, ≥ 1+, ≥ 20+), and include patients receiving ICI therapy.

## Conclusion

The 73 − 10 IHC represents a highly sensitive and specific approach for detecting PD-L1 in HNSCC, with minimal concerns about tumor heterogeneity and sampling bias. Our findings suggest that this method could expand the pool of eligible patients with HNSCC who are likely to benefit from ICI therapy. Further studies are needed to validate the clinical utility of the 73 − 10 clone and its potential as a predictive biomarker for ICI therapy in HNSCC.

## Electronic Supplementary Material

Below is the link to the electronic supplementary material.

Supplementary Material 1

Supplementary Material 2

Supplementary Material 3

Supplementary Material 4

## Data Availability

No datasets were generated or analysed during the current study.
