# Supplementary material for: High-Sensitivity PD-L1 Staining Using Clone 73−10 Antibody and Spatial Transcriptomics for Precise Expression Analysis in Non-Tumorous, Intraepithelial Neoplasia, and Squamous Cell Carcinoma of Head and Neck
Source: Head Neck Pathol. 2025 May 20;19(1):65. doi: 10.1007/s12105-025-01798-8 (PMC12092928; doi:10.1007/s12105-025-01798-8)
Supplement: Supplementary file 4 — Supplementary Material 4 [file 12105_2025_1798_MOESM4_ESM.docx]

**Article title:** High-Sensitivity PD-L1 Staining Using Clone 73-10 Antibody and Spatial Transcriptomics for Precise Expression Analysis in Non-Tumorous, Intraepithelial neoplasia, and Squamous Cell Carcinoma of Head and Neck

**Journal name:** Head and Neck Pathology

**Author names:** Yuri Noda^*^, Naho Atsumi, Takeo Nakaya, Hiroshi Iwai^3^, Koji Tsuta

**Affiliation and e-mail address of the corresponding author:**

Name: Yuri Noda

Affiliation: Department of Pathology, Kansai Medical University, 2-5-1 Shin-machi, Hirakata, Osaka 573-1010, Japan

E-mail: [nodayuridesu@yahoo.co.jp](mailto:nodayuridesu@yahoo.co.jp)


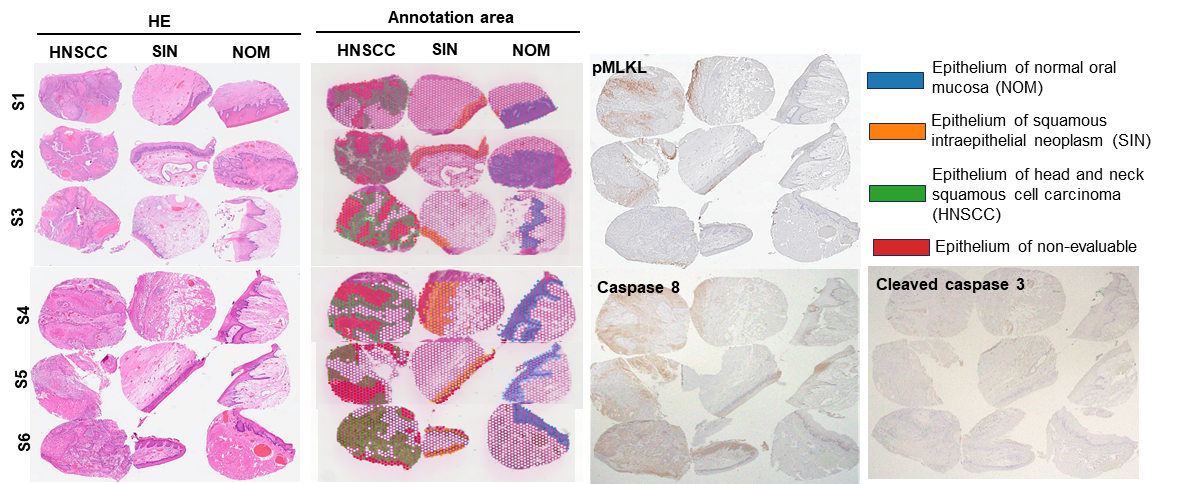


**ESM_3: Online Resource 3.** **Summary of the association area in Visium Spatial Transcriptomics and Differential Expression Analysis**

**Summary of the association area in Visium Spatial Transcriptomics**

1. First, annotaion of non-evaluable epithelium for 18 samples from 6 patietns [Sample (S) 1-6) was performed followed below immunohistochemical staining (IHC). As the death cells were non-evaluable for immunohistochemical analysis of imunocheck point inhibitor expression. Non-viable epithelium was as follows; morphologically lack the nuclei (p40 negative area), or shows positive staining for necroptosis marker such phospho-mixed lineage kinase domain-like (p-MLKL), or necrosis marker such cleaved caspase 3 (CC3) or caspase 8. For IHC analysis, tissue sections were incubated with antibodies against p40 (EPR17863-47, 1:1000 dilution; Abcam plc. Cambridge, UK), pMLKL (EPR9514; 1:50 dilution; Abcam plc. Cambridge, UK), CC3 (Asp175, 1:200 dilution; Cell Signaling Technology, Inc., MA, USA), and caspase 8 (ABM14C1, 1:200 dilution; Abcam plc. Cambridge, UK). For p40, pMLKL, CC3, and caspase 8, antigen retrieval was performed using ethylenediaminetetraacetic acid or citrate buffer at 95°C for 1 h, followed by detection using a Histofine SimpStain MAX-PO® polymer detection system (#NIC-414131F; Nichirei Bioscience Inc.), and visualization using diaminobenzidine.
2. Next, based on hematoxylin and eosin (HE) staining, annotation was performed on the epithelium of 18 samples. Non-evaluable regions identified in Step 1 were excluded, and annotations were applied to the normal oral mucosa (NOM), the epithelium of squamous intraepithelial neoplasms (SIN), and the epithelium of head and neck squamous cell carcinoma (HNSCC).

ESM_3: Online Resource 3. **Differential Expression Analysis**

In this study, differential gene expression analysis was conducted using scRNA-seq datasets to compare the expression patterns between different 6 samples (S1–S6) and three clusters (epithelial areas of NOM, SIN, and HNSCC). Analysis was performed using the Seurat package (v4.1.1) as follows:

**1. Data Preprocessing**

Quality control measures were applied to remove low-quality cells, based on the following thresholds:

- **UMI count**: Cells with fewer than 500 unique molecular identifiers (UMIs) were excluded.
- **Mitochondrial gene content (percentage mt)** Cells were excluded if their mitochondrial gene content exceeded the following sample-specific thresholds: S1,5%, S2,5%, S3,5%, S4,4%, S5,4%, and S6,5%.
- **Gene count (nFeature_RNA) and total RNA count (nCount_RNA)**: Cells were filtered using the following criteria:S1, S2 and S3 (nFeature_RNA: 500–9,000 and nCount_RNA: < 50,000) and S4, S5, and S6 (nFeature_RNA: 300–8,000 and nCount_RNA: < 40,000).

**2. Differential Gene Expression Analysis**

After transcriptomic data processing, differential gene expression analysis between the clusters and experimental conditions was performed using the FindMarkers function. The log_2_ fold changes obtained from the DEG were mapped onto KEGG pathways to visualize the pathways associated with differentially expressed genes.

**3. Statistical Analysis**

For differential gene expression analysis, the log-fold change threshold was set to 0.25, and p-values were adjusted using the Bonferroni correction to control for multiple testing. A minimum expression percentage (min.pct) of 0.1 was applied to identify significant differentially expressed genes in each cluster.

**
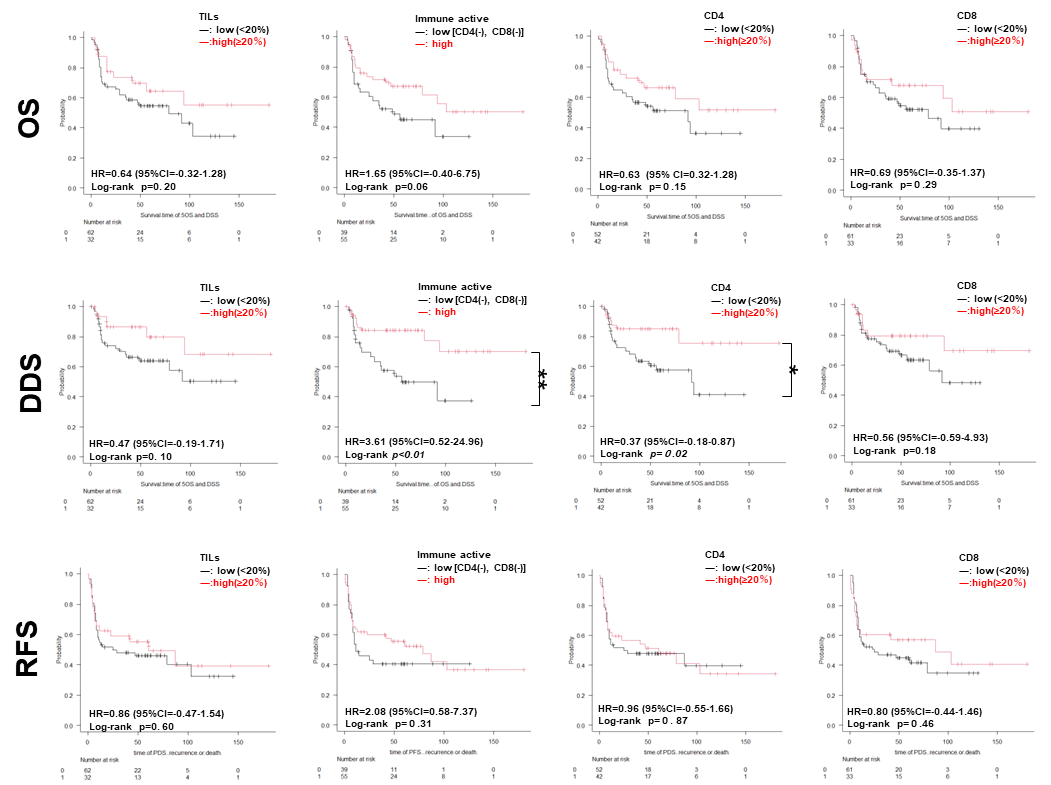
**

**Online Resource 4. Association between immune conditions of patients with HNSCC and their prognosis.**

To investigate the effects of immune activation on survival in patients with progressive HNSCC (n=94), CD4, CD8, TILs, and immunophenotypic features were analyzed. The log-rank test showed that low immune active [CD4(-) and CD8(-)], and CD4(-) HNSCCs are associated with a worse prognosis regarding DDS (*p* < 0.05), whereas CD4(-) HNSCCs, CD8(-) HNSCCs, HNSCCs with low TILs, and low immune active HNSCCs show trends toward negative effects on OS, DDS, and RFS (all *p* > 0.05) possibly due to the limited number of patients. CI, confidence interval; DSS, disease-specific survival; HNSCC, head and neck squamous cell carcinoma; HR, hazard ratio; OS, overall survival; RFS, recurrence-free survival; TIL, tumor-infiltrating lymphocytes.

| **CD274** |  | **vs NOM** | **vs SIN** | **vs HNSCC** | **vs all** |  |  |  |
| --- | --- | --- | --- | --- | --- | --- | --- | --- |
| S1 | **NOM** |  | -0.67494 | **-1.929608** | * |  |  |  |
|  | **SIN** | 0.6749401 |  | -1.254668 | * |  |  |  |
|  | **HNSCC** | **1.9296083** | 1.2546682 |  | **1.6345965** |  |  |  |
| S2 | **NOM** |  | * | -0.788383 | * |  |  |  |
|  | **SIN** | * |  | -0.616347 | * |  |  |  |
|  | **HNSCC** | 0.7883826 | 0.6163472 | * | 0.7464095 |  |  |  |
| S3 | **NOM** |  | * | -0.463025 | * |  |  |  |
|  | **SIN** | * |  | -0.455244 | * |  |  |  |
|  | **HNSCC** | 0.4630249 | 0.4552438 |  | 0.3588811 |  |  |  |
| S4 | **NOM** |  | -0.732933 | **-1.44803** | * |  |  |  |
|  | **SIN** | 0.7329328 |  | **-0.715097** | * |  |  | **log2** |
|  | **HNSCC** | **1.4480301** | **0.7150973** |  | **1.1176541** |  | 2.5 | **2.5** |
| S5 | **NOM** | * | -0.653787 | **-2.248151** | * |  | 1.25 | **1.25** |
|  | **SIN** | 0.6537875 | * | -1.594364 | * |  | 0.25 | **0.25** |
|  | **HNSCC** | **2.248151** | 1.5943635 | * | **1.8449118** |  | 0 | **0** |
| S6 | **NOM** |  | -0.371573 | -0.271909 | * |  | -0.25 | **-0.25** |
|  | **SIN** | 0.3715726 |  | * | * |  | -1.25 | **-1.25** |
|  | **HNSCC** | 0.2719085 | * |  | * |  | -2.5 | **-2.5** |

**Online Resource 5. The log2 folded value of *CD274* expression in six sets of NOM, SIN, and HNSCC samples.**

S, sample; HNSCC, head and neck squamous cell carcinoma; SIN, squamous intraepithelial neoplasia; NOM, normal oral mucosa.


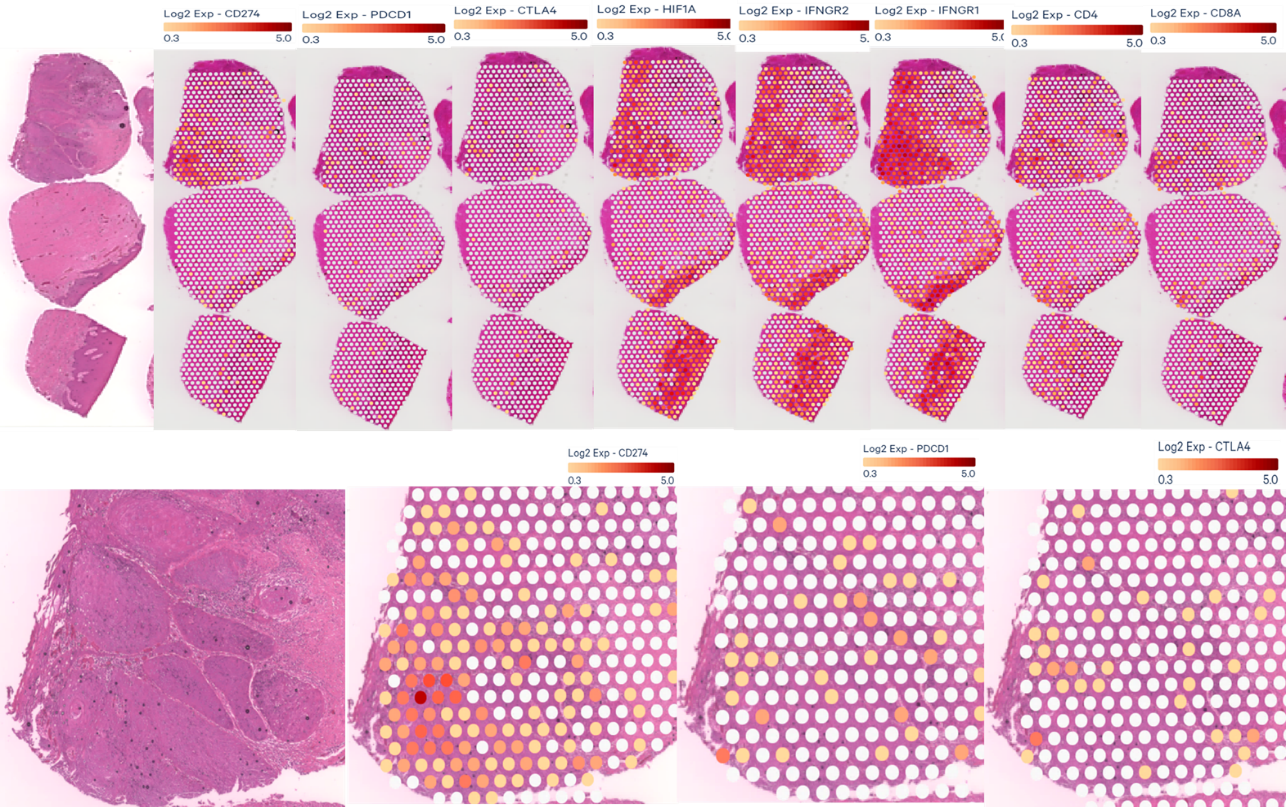
**Online Resource 7*.*** **The genes expression on Visium Spatial Transcriptomics associated with PD-L1 expression in cancers.**
